# Intermediate frequency of aversive conditioning best restores wariness in habituated elk (*Cervus canadensis*)

**DOI:** 10.1371/journal.pone.0199216

**Published:** 2018-06-25

**Authors:** Rob Found, Elsabé L. Kloppers, Thomas E. Hurd, Colleen Cassady St. Clair

**Affiliations:** 1 Department of Biological Sciences, University of Alberta, Edmonton, AB, Canada; 2 Banff National Park, Parks Canada, Banff, AB, Canada; Institute of Animal Science, CZECH REPUBLIC

## Abstract

In protected areas around the world, wildlife habituate to humans and human infrastructure, potentially resulting in human-wildlife conflict, and leading to trophic disruptions through excess herbivory and disconnection of predators from prey. For large species that threaten human safety, wildlife managers sometimes attempt to reverse habituation with aversive conditioning. This technique associates people as a conditioned stimulus with a negative, unconditioned stimulus, such as pain or fright, to increase wariness and prevent the need for lethal wildlife management. Resistance to aversive conditioning by some habituated individuals often results in more frequent conditioning events by managers, but there are few studies of conditioning frequency with which to evaluate the usefulness of this management response. We evaluated the effect of conditioning frequency on the wariness of elk (*Cervus canadensis*) by subjecting marked individuals to predator-resembling chases by people over a period of three months. In that time, animals were subjected to conditioning a total of 3, 4, 5, 6, 7, or 9 times which we analyzed as both an ordinal variable and a binary one divided into low (3–5) and high (6–9) conditioning frequencies. We measured wariness before, during, and after the conditioning period using flight response distances from an approaching researcher. During the conditioning period, overall wariness increased significantly for elk in both treatment groups, although the increase was significantly greater in individuals subjected to high conditioning frequencies. However in the post-conditioning period, wariness gains also declined most in the high-frequency group, equating to more rapid extinction of learned behaviour. Across all treatment frequencies, rapid changes in flight responses also characterized the individuals with the lowest wariness at the beginning of the study period, suggesting that individuals with greater behavioural flexibility are more likely to habituate to both people and their attempts to change wariness via aversive conditioning. Together, our results imply that aversive conditioning may be most effective at intermediate frequencies and that its utility might be further increased with proactive assessment of individual personalities in habituated wildlife.

## Introduction

Habituation describes the process by which individuals desensitize to stimuli that are encountered repeatedly without positive or negative consequences for fitness [1] [2]. Habituation by wildlife to people tends to occur wherever wildlife and humans share space, particularly where expanding human populations encroach on wildlife habitat, or in developed parts of protected areas where wildlife can escape both human hunting and natural predators [3]. Human-wildlife conflict with habituated wildlife is most likely to occur when it is accompanied by food-conditioning [4], such as when carnivores prey on livestock or pets [5] or herbivores target commercial crops and private gardens [6]. Examples of conflict-prone species include brown bears (*Ursus artos*) in Japan [7], elephants (*Elaphus maximus*) in India [8], dingoes (*Canis lupus dingo*) in Australia [9], and ungulates in the Americas [10] [11].

Even without food-conditioning, large species of habituated wildlife can threaten human safety and compromise ecosystem health. This is especially likely for ungulates, which habituate easily [12] and may abandon annual migratory routes to reside year-round in the predator refuges afforded by protected areas with high densities of humans [13] [14] [15] [16] [17]. Excessive herbivory in these predator refuges can degrade habitat and disrupt natural predator-prey cycles [18] [19] [20]. However, even when ecosystem impacts are severe, lethal management of hyper-abundant ungulates is unpalatable to the public, particularly for charismatic species residing in protected areas [21] [22].

As an alternative method to manage habituated ungulates, managers have attempted to treat them with aversive conditioning [23] [24] [25], which seeks to generalize to locations or contexts the response animals have to being frightened or ‘hazed’ (reviewed by[20]). Aversive conditioning employs associative learning through repeated exposures to paired stimuli by responding to an undesirable behaviour, such as a close approach to people, with an unconditioned aversive stimulus (reviewed by [26]), such as a predator-resembling chase by people or dogs [24]. If the desired association is learned and generalized, ungulates should be less comfortable around people and less likely to engage in conflict behaviour. Aversive conditioning has also been used to increase wariness in habituated black bears (*Ursus americanus*; [27] [28], but there are few published studies to guide the use of the technique. Consequently, managers attempt to employ aversive conditioning adaptively and reflexively, with varying intensity of stimuli, duration and frequency of treatment (personal observation). Unfortunately, this approach also makes aversive stimuli more predictable in time or space, which usually reduces its efficacy [29]. Moreover, a gradual increase in the intensity of any punishment tends to cause habituation, not the intended effect of sensitization [26]. Perversely, wildlife may become habituated to the very treatments that were intended to reverse their habituation and instill greater wariness. Finally, escalating stimuli are both expensive and ethically difficult to defend. More systematic study of the factors that increase the efficacy of aversive conditioning for wildlife could provide a needed alternative to lethal management with the least cost to humans and wildlife.

The objective of this study was to examine the effect of one factor, exposure frequency, on the efficacy of aversive conditioning as a means of increasing wariness in habituated elk in the townsite of a Canadian mountain park. We assessed the wariness of individual elk using two dependent variables: flight response distance [12] [30] and daily proximity to a town site boundary. We chose exposure frequency as our primary independent variable to complement a prior study of treatment type [24] and because it has large impacts on treatment costs (through the associated labour by people) and animal ethics (through repeated collateral harassment of non-targeted elk). Most importantly, we also considered the sustainability of wariness gains, as measured by declining wariness behaviour after conditioning is stopped (i.e. recidivism; sensu [31]).

**Fig 1:**
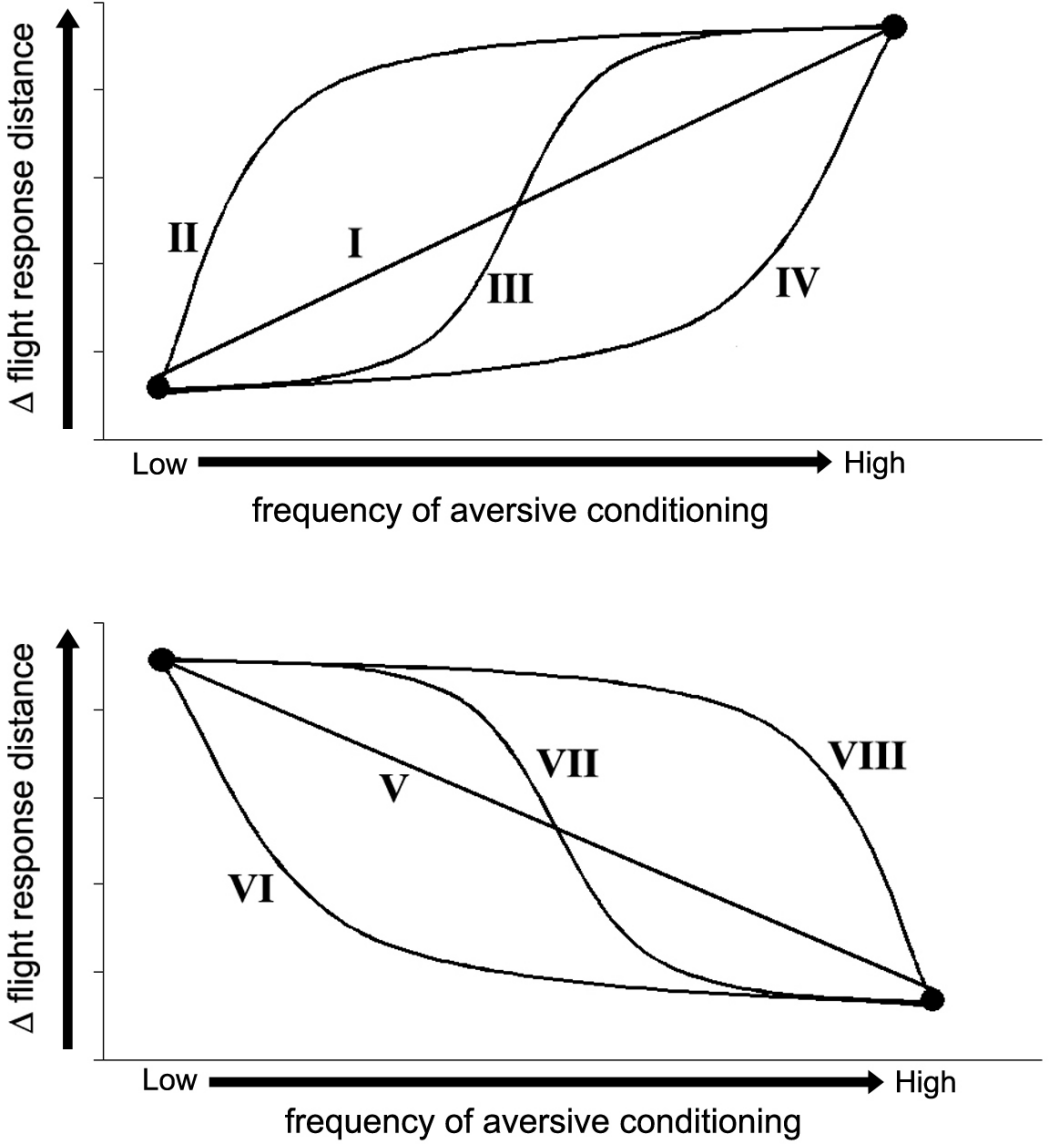
Hypothetical curves for changes in elk flight response distance at different frequencies of aversive conditioning, (top) *during* the conditioning period, and (bottom) *after* the conditioning period. Curves illustrate: (I) a linear response where flight distance increases at constant rate with increasing conditioning frequency, or threshold responses whereby flight distance increases rapidly after a threshold is reached at conditioning frequencies that are (II) low, (III), moderate, or (IV) high. Comparable relationships could occur in the post-conditioning period to illustrate the rate at which flight response distances decline with thresholds (Curves V–VIII) triggered at different conditioning frequencies.

Based on the implicit expectations of local wildlife managers, learning theory, and our own previous work with aversive conditioning of elk [24], we predicted that wariness would increase with conditioning frequency, but we were unsure whether that change would occur in a linear or logarithmic way (Fig 1A). Because laboratory studies have shown that conditioning frequency affects the rate at which animals both learn and abandon new behaviours [32] [26], we predicted that wariness would decline more rapidly after the cessation of conditioning at higher frequencies, which could also occur either linearly or logarithmically (Fig 1B). As an average of these tendencies, we predicted that intermediate conditioning frequencies would maximize gains in conditioned wariness while minimizing recidivistic losses of wariness once conditioning was over. We also speculated that wariness would vary with some consistency among individuals, but independent of treatment (i.e., past experience or as a personality trait, following [17].

## Materials and methods

Fieldwork was conducted in the townsite and surrounding area of Banff, AB (51°15’N, 116°30’W), within Banff National Park, Canada. The townsite has a permanent human population of 7500 people, but temporary park visitation approaches five million people per year [33]. Banff townsite is situated in the Bow Valley, within the central Canadian Rockies, at an elevation of 1383 m. The Bow Valley bottomlands typically have modest snowfall combined with occasional warm periods, creating important winter habitat for ungulates. Our study area consisted of the urban land-use area of Banff and adjacent montane wetlands, forests, and shrublands [34], plus a golf course 2 km from the town boundary, for a total area of 466.5 ha. The area is bisected by a major east-west highway that is mitigated with a wildlife exclusion fence and periodic crossing structures that support movement across the highway by elk and other animals [35].

We conducted the field work for this study in winter 2002–2003, which followed a spring census of 137 elk in the town site area with herds typically composed of adult females with young-of-year and yearling offspring of both sexes (Banff National Park, *unpublished data*). We conducted the study in winter when habituated animals congregate in the townsite and when there are fewer seasonal influences on elk behaviour (e.g., mating or calving). We focused our conditioning treatments on resident elk (determined via radio-telemetry conducted by Parks Canada staff in summer) that occur in or near the townsite year round, but occurred during our study as a single large herd that moved freely throughout the Banff townsite and periphery. Thus, we expected that elk from the different treatment groups (below) were subjected to relatively similar forage availability, habitat, predation pressure and other environmental factors that could influence our dependant variables, such as the presence of wolves (*Canis lupus*) described below.

We based our study on 25 adult (> 2 years) female elk that could be individually identified by their ear-tags and radio collars. Because elk are captured in the park by live-darting, all the elk used in our study were habituated to the extent that we could generally approach each to within 40 m. This familiarity made it possible for us to record the position of most study elk daily. We assessed both habituation level at the start of the study and individual responses to conditioning (below) with flight response distance. This variable is the distance at which an animal moves away from an approaching human [24] [36]. All captures, aversive conditioning treatments, and flight response measures were conducted in accordance with University of Alberta Animal Care Committee Permit #354111.

### Aversive conditioning treatments

We divided the 25 elk into five treatment groups based on intended frequencies of conditioning events each 10, 15, 20, 25 and 30 days, to generate between 3 and 9 conditioning events per individual over the 90 day conditioning period. Previous work showed that the highest frequency (i.e., a conditioning event each 10 days) was sufficient to achieve management objectives for wariness (24). Based on flight response distance before conditioning began, we assigned elk to treatment groups to generate similar averages, thereby avoiding unintended differences among groups in initial wariness. Variation in elk position and availability for treatment caused us to deviate from the five intended categories of conditioning frequency to produce a final distribution of six frequency groups (3, 4, 5, 6, 7 or 9 conditioning events), containing 7, 7, 5, 1, 1, 3 individuals, respectively (one elk was largely unavailable for conditioning and so excluded from analysis). In our analyses (below) we used these actual trial frequencies as a continuous independent variable, but also condensed them into frequency categories above and below the median frequency, which we called low (3–5 trials per elk; N = 20 individuals) and high (6–9 trials per elk; N = 5 individuals). This binary categorization overcomes the difficulty we had in perfectly matching our intended conditioning frequencies and provides information that may be easier to generalize to other jurisdictions.

We applied aversive conditioning treatments (below) to each elk, between December 2002 and February 2003, in relation to their designated frequency schedule. We measured flight responses immediately before each trial. Elk were only conditioned when they were found within the town site or on peripheral entry points to it. Because elk of different treatment groups were generally interspersed within herds, a treatment was only applied if the target individual could be split away from the others using gentle herding techniques adapted from cattle management [37] [24]. This approached confined the bulk of the aversive stimuli to the target elk only, which was desirable both as an experimental control, but also ethically desirable for reducing unintended aversive stimuli of non-target individuals. Although this procedure resembles conditioning–and thus could either sensitize or de-sensitize elk to approach by people—it was done consistently for all treatment groups. If it was not possible to separate elk using gentle herding methods at a given time, we left the herd alone, and did not proceed with that scheduled conditioning trial on that elk. In some cases this meant we had to adjust the assignment of individuals to categories to accommodate such deviations from the intended frequency schedule, but we ensured that the mean flight response distances before conditioning remained equal between treatment groups.

The aversive conditioning treatments followed a protocol based on human conditioning that successively increased flight responses distances previously [24]. In brief, elk were chased for 15 minutes by two people on foot that each simultaneously waved a hockey stick above their heads. Strips of crisp plastic flagging tape were tied to the distal end of the stick which rattled in the wind to create a visual and auditory stimulus. Elk were chased in a direction away from the remaining herd and the town site, and towards suitable grazing habitat. If vehicular traffic, infrastructure, or people compromised elk or human safety, elk were gently herded towards the edge of town before the chase was begun, or the trial was abandoned entirely. During the conditioning trials we moved the animals as far and as quickly as possible, typically at a running pace, and we used snow tracking to maintain pursuit if elk moved temporarily out of sight. All trials were tracked with a handheld global positioning system (GPS; Trimble GeoExplorer3; Trimble, Sunnyvale, California) carried by the person undertaking the chase to measure the distance elk were displaced.

We targeted the schedule of conditioning to achieve the frequencies described above (one event per 10 to 30 days, or 3–9 events total), which necessitated some opportunistic conditioning events to make up for missed events. A minimum of 24 hours separated all conditioning events for a given individual. We collected and averaged flight response distances as pre-conditioning *before* data from September through November, conditioning *during* data from December through February, and post-conditioning *after* data from March through April. We described the change in flight responses distances achieved between periods as the ‘conditioned change’ (*before vs*. *during*), recidivism'‘ (*during vs*. *after*) and the ‘overall change'‘ (*before vs*. *after*).

### Dependent variables and statistical analyses

Flight response distance was measured only when the target elk was neither bedded down nor travelling, which we defined as a steady pace in a consistent direction, and was at least 25 m away from vegetation that was sufficiently large and dense to conceal an elk. One person approached the focal elk at walking speed, and with a neutral posture, from a start distance of >75 m and recorded the closest distance the elk could be approached before it reacted by moving at least 5 m in any direction. We used several trained observers, who changed clothing between trials, to minimize habituation to researchers. All flight response measures were taken opportunistically throughout each of the *before*/*during*/*after* conditioning periods, depending on where and when elk were encountered in situations fitting the above criteria. However, *during* the conditioning period flight response measures were taken at least 2 days after the last conditioning event. Each individual had its flight response distance measured a minimum of 3 times (mean = 4.9) *before*, a minimum of 6 times (mean = 10.0) *during* the conditioning period, and 3 times (mean = 4.4) *after* the conditioning period. All distances were measured with a digital range finder (Bushnell Yardage Pro 500; Bushnell Corporation, Overland Park, Kansas), accurate to within 0.5 m.

Because proximity of wolves influences elk behaviour [38] [39] [40], we calculated a relative index of wolf pressure *post hoc* using tracking data from wildlife underpasses near the town site (A. Clevenger, *unpublished data*). Wolves and other predators access the town from the north primarily by using these underpasses. As an index of wolf activity, we averaged the number of wolf southbound passages (towards the town site) for the week preceding each flight response trial.

We defined the town site boundary with an imaginary line that encompassed the outer edge of all associated human infrastructure and conducted conditioning only within this area. To assess changes in elk distribution in response to conditioning, we recorded one daily morning visual sighting or radio-telemetry location of each marked elk throughout the *before*, *during* and *after* conditioning periods. Elk locations were recorded prior to measurements of flight response or the application of conditioning treatments. From those locations, we calculated the distances between individual elk and the nearest position of the town site boundary, using ArcGIS Spatial Analyst (Environmental Systems Research Institute, Redlands, California, USA). We averaged the distances for each elk within the conditioning periods of *before*, *during* and *after* (as defined above).

We conducted three analyses to determine the effect on elk flight response distances of differences in the frequency of conditioning events (Table 1). First, we used t-tests to compare mean flight response distances of elk in the low versus high conditioning frequency groups (above), within each of the *before*, *during*, and *after* conditioning periods.

**Table 1:** Model structures and variables for each of three different types of analyses of the effects of aversive conditioning frequency, and its subsequent removal, on elk flight response distance or proximity from town as the result of conditioning, and the effect of habituation level (pre-conditioned flight response distance) on changes to flight response effect sizes during and after conditioning.

	Dependent variable	Independent variables
Analysis 1 (t-test)	Mean flight response distance in each of before / during / after periods	High vs. Low conditioning frequency
Analysis 2 (Generalized linear fixed effects model)	*Change* in • flight response distance or • proximity to town boundary For two periods • Conditioning (before-during) • Recidivism (during-after)	• Trial frequency • Group size • Distance to neighbor • Distance to cover • Location within group (binary) • Snow depth* • Wolf presence*
Analysis 3 (Generalized linear mixed effects model)	Change in flight response in relation to individual variation In each of • Conditioning (before-during) • Recidivism (during-after)	• Conditioning frequency (random) • Average initial flight distance (fixed)

*Only for proximity measure

In a second analysis, we used generalized linear fixed effects models (GLM) to examine changes in each of flight response distance and proximity to the townsite boundary between successive conditioning phases. We built these models using purposeful selection of variables (following [41]), using *P* < 0.25 for inclusion in the model, *P* < 0.10 for retention, included all confounding variables (i.e. those influencing the parameters of any remaining variables by more than 20%) and then tested each ecologically-relevant, two-way interactions among variables. We tested each candidate model to determine if a linear or quadratic form improved the model fit, then used Akaike's Information Criterion to select the best-fitting model for each phase of conditioning: Conditioning (where the dependent variable was for the conditioned change from Before to During) and Recidivism (where the dependent variable was for the response change between the During and After stages of the conditioning). We modeled the change in flight response distance for each of these periods as a function of actual trial frequency (i.e. a total of 3, 4, 5, 6, 7, or 9 events occurred over the 90-day conditioning periods) and four additional variables: group size ($\bar{x}$ = 50.5 ± 1.42 elk), distance to nearest neighbour ($\bar{x}$ = 6.8 ± 0.30m), distance to cover ($\bar{x}$ = 56.5 ± 1.97m), and location within the group (interior vs. peripheral). A group was defined as a discrete gathering of elk that appeared to make uniform decisions to move, graze, or bed down. Elk on the edge of a single group were classified as being in one of two positions; peripheral if they were on the edge of the herd or interior if they were surrounded by other elk (after [17]). Models for elk displacement from the townsite boundary included two additional covariates: average snow depth ($\bar{x}$ = 16.6 ± 0.54cm) and wolf presence (index of wolf track crossings at underpasses: $\bar{x}$ = 12.9 ± 0.46).

In a third analysis, we modelled the change in flight distances between each period (as above, before to during = conditioned change; during to after = Recidivism), but depicted these differences as effect sizes, added the average of the *before* flight response distances for each individual as a fixed effect, and included actual conditioning frequency (measured as above) as a random effect. The purpose of these generalized linear mixed effects models was to better understand how initial wariness might affect responses to conditioning. By describing changes in flight responses as effect sizes rather than absolute values, we could avoid obscuring the importance of relative individual changes for elk with different levels of habituation (i.e. a 10 m change is more meaningful in an elk with an initial flight response of 10 m, compared to an elk with an initial flight response of 50 m).

Owing to small sample sizes, we set *alpha* = 0.10 in all analyses to balance Type I and II errors. All analyses were conducted using Stata 11.1 software (Statacorp).

## Results

Each elk received 3 to 9 conditioning treatments (***x*** ± SE = 4.8 ± 0.14, ***N*** = 25) over the winter season, which we divided into *post-hoc* categories of low (3–5 events) and high (6–9 conditioning events). There was no difference in the proportion of time that individuals spent with elk from their own group (***x*** ± SE = 65.7 ± 8.9%) in comparison to elk from the other group (61.7 ± 4.3%; *t*_1,71_ = 0.62, *P* = 0.43).

### Effects of conditioning frequency on flight response changes

**Fig 2:**
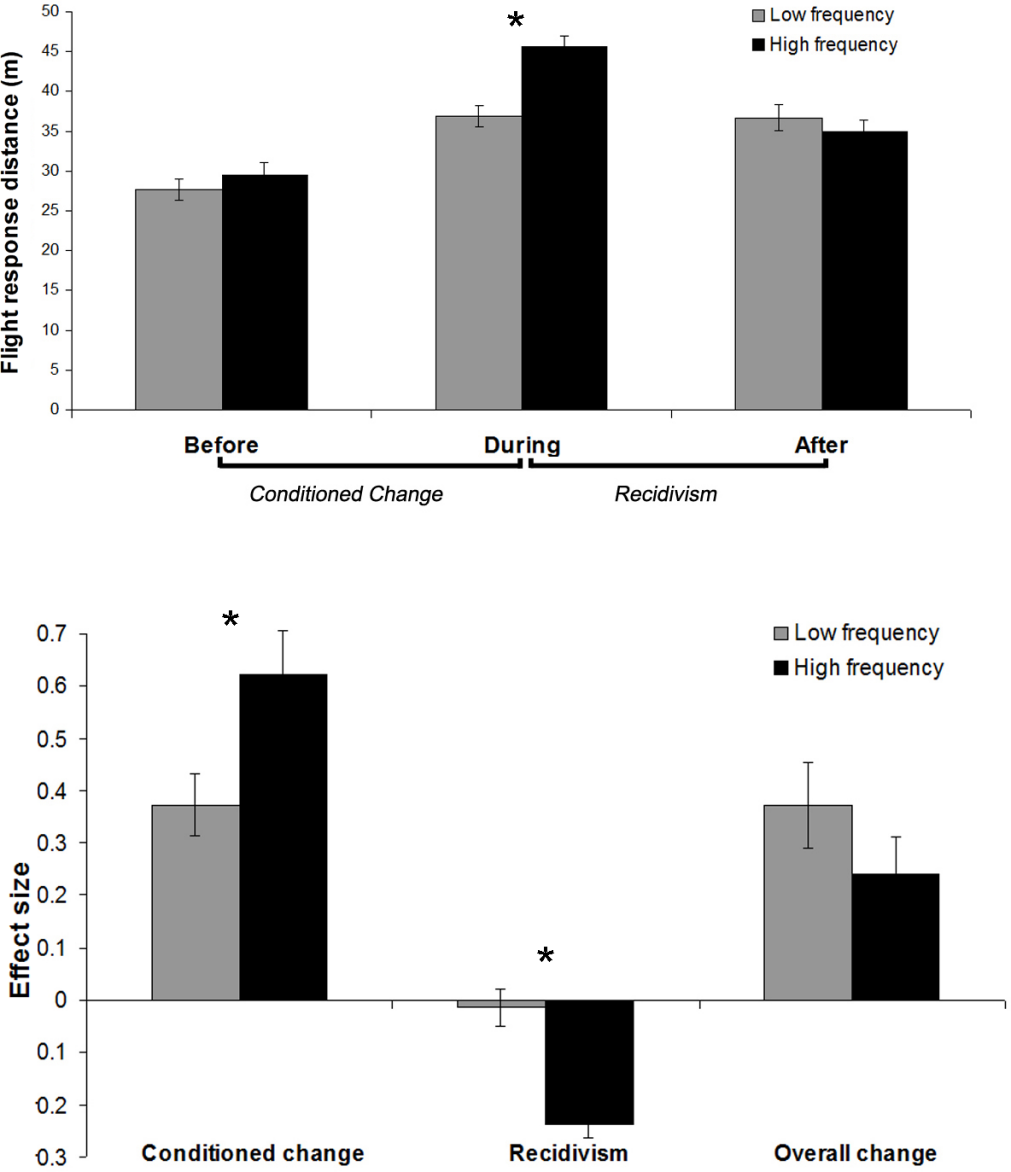
(top) Average flight response distances for individual elk in periods *before*, *during*, and *after* a 90 day aversive conditioning period. Low frequency is defined as 3–5 total aversive conditioning treatments, high frequency as 6–9 total treatments. Difference between *before* and *during* flight responses is the conditioned change", and difference between *during* and *after* flight responses is recidivism. Effect sizes for these behavioural changes, and the overall change from *before* to *after*, are shown in the bottom panel of the figure.

Relative to the period *before* conditioning began, elk in the high frequency group increased their flight responses *during* conditioning by almost twice as much (62.2% to 45.6 m ±1.52 m) as elk in the low frequency group (37.3% to 36.9 m ±1.29 m; *t*_24_ = 2.64, *P* = 0.015; Fig 2). Averaged over treatments of varying conditioning frequency, elk exhibited 30% higher flight response distances *after* conditioning (38.7 m ± 1.5 m), compared to *before* conditioning (28.1 m ± 1.3 m; *t*_44_ = -5.40, *P* < 0.001). In the 8 weeks *after* conditioning was completed, average elk flight response distances subsequently declined by 6.7% (36.27m ± 1.6 m; t_45_ = 1.11, *P* = 0.27). The magnitude of this change was dependent on conditioning frequency. In the low frequency group, flight responses declined by only 1.2% (0.8 m ± 1.4 m), but in the high frequency group, it declined by 23.5% (10.7 m ± 2.6 m; t_21_ = -2.62, *P* = 0.016; Fig 2). Because of this recidivistic effect, the overall conditioned change (in the *before* to *after* periods) was a 37.3% reduction in flight response in the low frequency group (-8.8 ± 2.4 m; t_34_ = -3.60, *P* = 0.0010) and a 24.1% reduction in flight response in the high frequency group (- 5.4 ± 5.0 m; t_8_ = -1.09, *P* = 0.31).

**Fig 3:**
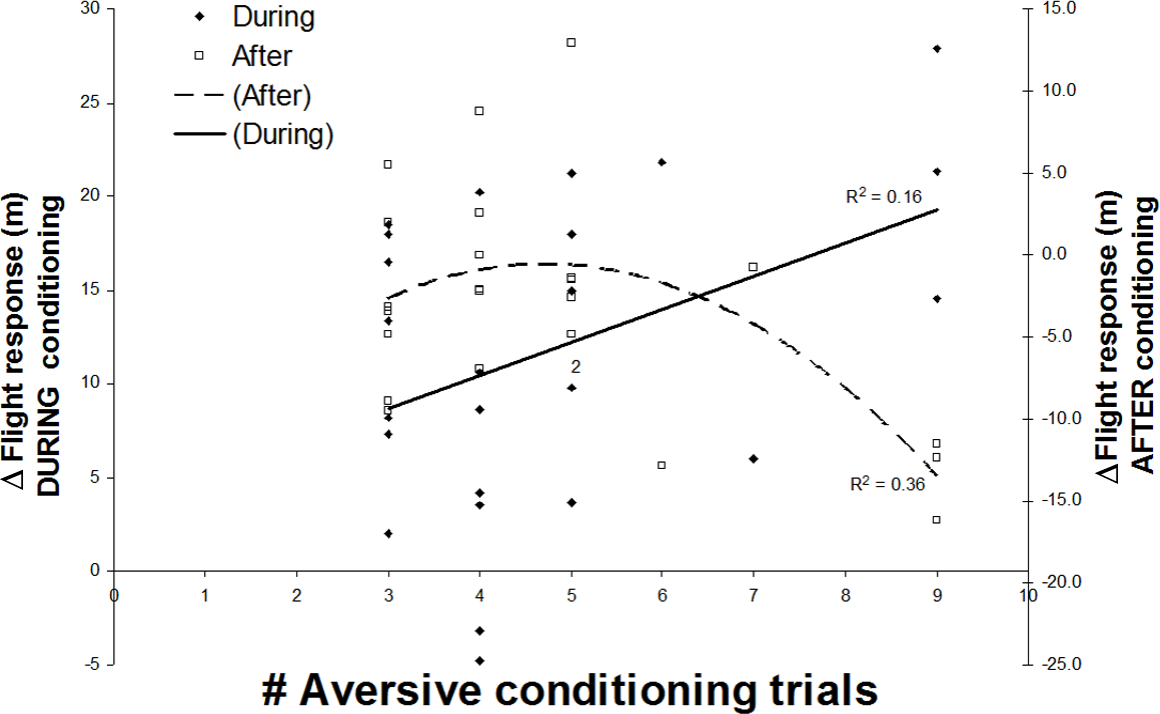
Change in flight response distances for elk exposed to different frequencies of aversive conditioning, contrasting the changes that occurred between *before vs*. *during* (conditioned change) and *during vs*. *after* (recidivism).

Flight response distances in the *before* period were not predicted by any of nearest neighbour, distance to nearest cover, position within the herd, group size, or assignment to frequency group, in either univariate or combined models (F_1,22_ ≤ 0.74, *P* ≥ 0.40, r^2^ ≤ 0.034). The best-fitting model for the change in mean flight response distance from *before* to *during* contained only the absolute measure of conditioning frequency (z _21_ = 2.05, LL = -77.45, *P* = 0.041), with a quadratic fit to the data (Fig 3). No other covariates were significantly correlated with this conditioned change (F_1,22_ ≤ 2.08, *P* ≥ 0.16, r^2^ ≤ 0.090). The best-fitting model for the recidivism response (i.e. between *during* and *after*) response also only contained trial frequency (z_21_ = 2.30, LL = -74.9, *P* = 0.022), also best fit as a quadratic term (Fig 3). No other covariates were correlated with this recidivistic change in flight response distance (F_1,22_ ≤ 0.65, *P* ≥ 0.26, r^2^ ≤ 0.060).

### Effect of individual level of habituation

**Fig 4:**
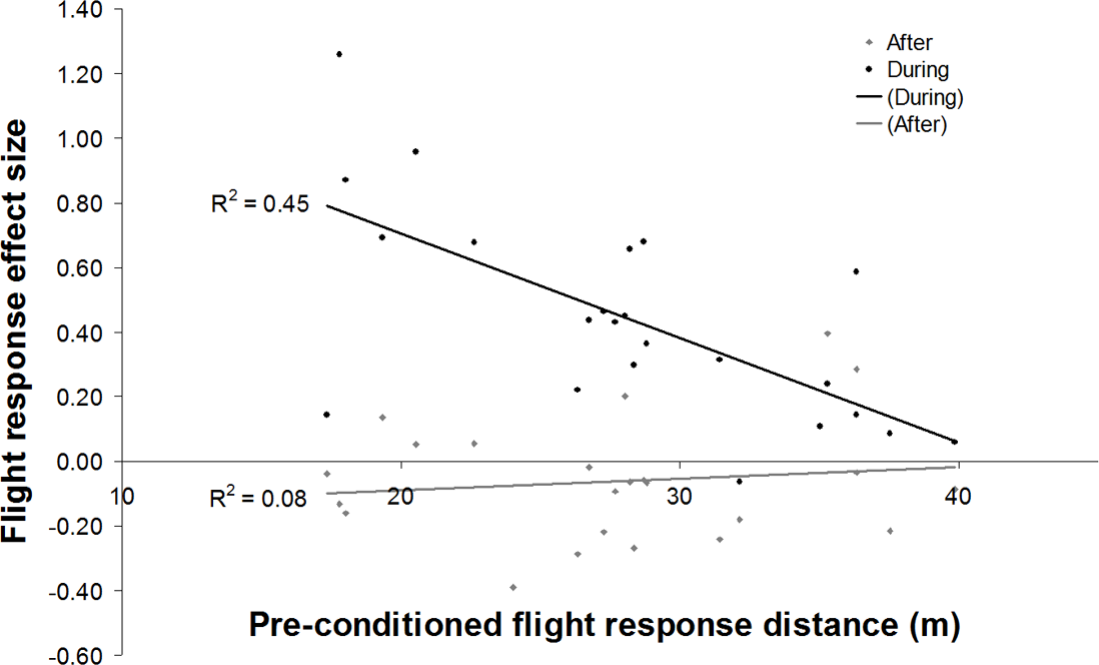
Effect of habituation, measured as the average flight response distance measured *before* conditioning (X-axis). Effect sizes (Y-axis) are for changes in mean flight response distances *during* aversive conditioning and *after* conditioning stopped.

Habituation level, as measured by individual mean flight responses *before* conditioning, was correlated with flight response changes *during* conditioning (F_1,22_ = 16.90, *P* < 0.0001, R^2^ < 0.446; (Fig 4). However, habituation level was not significantly correlated with the change in flight response *after* conditioning (F_1,22_ = 1.90, *P* = 0.18, R^2^ = 0.083; Fig 4). Habituation level was also significantly correlated with the overall (net) change in flight response from *before* to *after* (F_1,22_ = 19.37, *P* < 0.001, R^2^ = 0.480).

When we modelled conditioning frequency as a continuously-distributed random effect in the conditioned change (*before vs*. *during*) in flight response distances, we found positive effects of both habituation level and peripheral positioning within the herd (χ^2^_17_ = 33.46, LL = -2.43, *P* < 0.001; habituation, z = -5.16, *P* < 0.001; position, z = 2.77, *P* < 0.01). For the subsequent period of recidivism (*during vs*. *after*), the best-fitting model included only group size (χ^2^_17_ = 3.69, LL = 0.469, *P* = 0.055). In the model of overall change (before *vs*. after), a significant interaction between conditioning frequency and habituation level revealed that more habituated elk had greater declines in flight response distances (χ^2^_17_ = 19.37, LL = -8.01, *P* < 0.001). Wolf activity did not influence elk responses to aversive conditioning in any conditioning period or at any conditioning frequency.

### Proximity to town site during and after conditioning

During conditioning trials, we displaced elk an average distance of 918 m (± 42.5 m).

Based on the daily positions of individual elk, the average distance between elk and the Banff town boundary increased by 43 m (± 51 m) *during* aversive conditioning (t_44_ = 0.422, *P* = 0.67). The best-fitting model for this displacement *during* conditioning included only the elk position, wherein peripheral elk were displaced further from the townsite boundary (F_1,21_, *P* < 0.01, R^2^ = 0.31). As univariate effects, trial frequency was also significantly correlated with displacement *during* conditioning (F_1,21_ = 4.38, *P* = 0.049, R^2^ = 0.17), but habituation level was not (F_1,21_ = 0.35, *P* = 0.56, R^2^ = 0.016).

Average elk distance from the Banff town boundary increased an additional 135 m (± 50 m) *after* conditioning (t_44_ = -1.35, *P* = 0.19), for an overall displacement from town of 178 m (t_44_ = -1.39, *P* = 0.17). The best-fitting model for this displacement did not include either trial frequency or habituation, but showed displacement was positively correlated with increasing distance to nearest neighbour (z_20_ = 2.44, *P* = 0.015) and peripheral positions within the herd (z_20_ = 9.22, *P* < 0.001). Models with only trial frequency (F_1,21_ = 0.01, *P* = 0.93) or only habituation level (F_1,21_ = 0.64, *P* = 0.43) were not correlated with displacement from town *after* conditioning.

## Discussion

Our results suggest that aversive conditioning can significantly increase elk wariness of humans, but that the effectiveness of both the learning and extinction of these behavioural changes is dependent on the frequency of conditioning events. Clearly there must be a minimum effective frequency for conditioning (even if that minimum is just one conditioning trial), but our study showed that there is also a maximum effective frequency. Individual elk in our study that were exposed to high-frequency aversive conditioning demonstrated the largest increases in wariness during the conditioning period, but these same individuals also showed the greatest recidivism once conditioning was stopped. Interpolating this difference suggests that elk exposed to intermediate frequencies of conditioning balanced conditioned gains with recidivistic losses such that they exhibited the largest net increases in wariness to humans over the duration of our study. These learned responses were further influenced by the level of habituation shown by each individual elk before the study began. The most habituated individuals were significantly more responsive to conditioning, but also showed slightly more recidivistic behaviour once conditioning ceased.

Predicting animal learning responses to different conditioning frequencies is important for the utility of aversive conditioning as a management tool. We predicted that elk flight response distances would increase during the conditioning period, following one of several potential curves (Fig 1). Our results were most congruent with the linear responses predicted by curve "I", for which changes in flight response were expected to continue increasing with no apparent peak or plateau. While this seemingly predicts that wariness would continue to increase at even higher conditioning frequencies, results from the post-conditioning period suggest that these wariness gains would likely be lost through even higher rates of recidivism. The recidivistic behaviour demonstrated by our study animals was most similar to our predicted curve "VIII", in which recidivism did not much occur at a low conditioning frequency, but increased sharply as conditioning frequency increased.

These paired results, showing that a greater frequency of aversive conditioning increased both learned responses in the short term and the extinction of those responses when the aversive stimuli were removed, are consistent with previous research on associative learning in other animals. Laboratory studies have shown that higher frequencies of conditioning can render learned associations more routine and expected, and thus make the subsequent removal of conditioning stimuli more readily apparent [26]. This interval length between training sessions is also important for memory consolidation, and so continuous increases in trial frequency are not expected to result in continued behavioural changes [42]. While our results did not predict such a plateau, one may still exist at an even higher hypothetical conditioning frequency.

Learning theory further posits that ongoing rewards or punishment should occur unpredictably to prevent the extinction of many learned behaviours [43]. For example, [44] found that retention of non-aversive (reward-based) instrumental training was improved for dogs treated at intermediate frequencies, while [28] found that black bears (*Ursus americanus*) conditioned at a lower frequency were less likely to continue visiting human-dominated areas compared to bears conditioned at a higher frequency.

The optimal frequency for an aversive conditioning regimen must balance conditioned gains with recidivistic losses. While small sample sizes limited an explicit demonstration of this effect, statistical models derived from responses by elk in our study suggested this balance was reached at an intermediate level of around 6–7 conditioning trials over 90 days, or around one event every two weeks. Besides resulting in the greatest overall gains in wariness behaviour, this intermediate conditioning frequency may also support a more ethical and affordable use of this management tool. In Banff National Park, previous conditioning strategies were highly predictable in time and space. This may have actually increased habituation and produced elk that had become unresponsive to human approaches, resulting in the eventual need for lethal management (T. Hurd, *personal observation*). Similarly, seemingly counter-intuitive outcomes have occurred elsewhere. For example, 6 of 11 bears conditioned at high frequency in Yosemite National Park were eventually killed or relocated because conditioning was ineffective, and the bears became too aggressive to people [28]. Avoiding excess conditioning also prevents stressing animals unnecessarily, and via collateral effects on non-targeted individuals.

In addition to the variation in behavioural responses between different conditioning frequency classes, we found substantial variation among individual responses within classes. Elk exhibiting the least wariness before conditioning began exhibited the greatest subsequent increases in wariness during the conditioning period. In this respect, our results appear to differ from those found by [28], who reported that habituated black bears were more resistant to aversive conditioning than wilder bears. However, they are consistent with some subsequent work of our own in which elk with bolder personalities were more likely to exploit human-disturbed landscapes [17]. These animals were also more responsive to both the onset of conditioning and its removal [45]), and exhibited more behavioural flexibility [46].

While recidivistic change was not correlated with habituation level in our study, the capacity to learn may be a personality trait in elk, and individual variation in behavioural flexibility may result in some individuals that are more prone to habituation than others. Variations in hormones between sexes, but also within sex cohorts, resulted in variation in learning and retention of conditioned fear responses in laboratory rats [47]. Personality variation in humans has been correlated with responsiveness to conditioning [48]. Proclivities to habituate and respond to aversive conditioning may be components of elk personality, and if personality correlates could be identified, this would enable pre-emptive conditioning that could prevent the negative ecological and trophic consequences of habituation behaviour before they ever occur. We found that conditioning impacted wariness across all levels of individual habituation, but more studies may be needed to disentangle the effects of individual variation in wariness from those caused by conditioning frequency. Meanwhile, managers could benefit from greater awareness of the advances in understanding individual variation in other contexts, particularly as it relates to personality and behavioural syndromes [49].

Separating the variation stemming from management regimes (e.g., conditioning frequency) and individual variation is challenging in free-living animals where behavioural response are also influenced by a host of social factors. The failure of aversive conditioning to demonstrably displace elk further from the townsite boundaries is likely the result of group cohesion, which would counteract the effects of increases in wariness by individuals. Elk are highly gregarious ungulates, and the behaviour of entire herds was likely affected during and after each conditioning event. Herding animals are likely to respond to aversive conditioning by seeking protection via tighter associations with other individuals and more advantageous positions within groups [50] [51]. More generally, all social vertebrates are probably more susceptible to group effects of aversive conditioning. For example, wolves (*Canis lupus*) responded as a pack to avoid particular areas where some pack members were exposed to aversive stimuli [52]. We found that elk group size was positively correlated with recidivistic changes in wariness, indicating some degree of social learning regarding responses to humans, and may thus implicate social behaviour in the process of habituation itself.

In addition to the herd-based dynamics of social animals, the effects of aversive conditioning may be countered or exacerbated by the perceived risk of predation from natural predators. In our study, elk behaviour did not appear to be altered by wolf activity, which was extremely low during our study period. The distribution of ungulates relative to townsites can change significantly in response to both natural predators [38] and aversive conditioning [53], because ungulate use of townsites is a balance of risks from predators outside of the townsite, and humans within it. We found that elk distance from town increased slightly, but not significantly, during conditioning, and was unaffected by conditioning frequency or habituation level.

We showed that the frequency of treatment can affect both immediate and longer-term responses to aversive conditioning, and that an intermediate frequency appears to best balance the ability of this management tool to achieve–and then maintain–desired increases in wariness. In general, aversive conditioning is more likely to increase wariness of individual than to change their distribution (for example, in relation to exclusion zones). That effect is likely sufficient to increase public safety in contexts where danger is posed by close proximity of people and ungulates, as opposed to the presence of ungulates in areas that are sometimes frequented by people. More research on the application of aversive conditioning and related forms of associative learning is needed to counter the increasing rates of wildlife habituation and its deleterious effects on ecological integrity, particularly in jurisdictions where lethal management is unacceptable to the public or incompatible with conservation goals [51] [29], Such research could also apply to additional conservation contexts, such as the reintroduction to the wild of captive-bred populations [54], and the intentional habituation of species targeted by eco-tourism as a method of garnering conservation funding [55]. In each of these contexts, there is also an urgent need to better understand the sources of variation among individuals so as to maximize the beneficial outcomes of learning-based interventions.
